# Evaluation of Crack Formation and Propagation with Ultrasonic Root-End Preparation and Obturation Using a Digital Microscope and Optical Coherence Tomography

**DOI:** 10.1155/2019/5240430

**Published:** 2019-12-30

**Authors:** Bayan Rashed, Yoshiko Iino, Arata Ebihara, Takashi Okiji

**Affiliations:** ^1^Department of Pulp Biology and Endodontics, Graduate School of Medical and Dental Sciences, Tokyo Medical and Dental University (TMDU), Tokyo, Japan; ^2^King Abdul-Aziz Airbase Hospital, Dhahran, Saudi Arabia

## Abstract

**Objective:**

This study is aimed at determining (1) the effect of root-end resection, ultrasonic root-end preparation, and root-end filling on the incidence of crack formation and propagation by using a digital microscope (DM) and optical coherence tomography (OCT) and (2) the performance of OCT on the detection of cracks by comparing with microcomputed tomography (micro-CT) as a reference standard.

**Methodology:**

Thirty extracted lower incisors were endodontically treated and subjected to root-end resection and ultrasonic root-end cavity preparation. Then, the teeth were divided into three groups (*n* = 10, each), and the root-end cavity was either left unfilled or filled with mineral trioxide aggregate (MTA) or super-EBA. The resected surface was observed with OCT and DM after the root-end resection, ultrasonic root-end preparation, and root-end filling, and the frequency of incomplete and complete cracks were recorded. The observation was repeated after two weeks, one month, and two months, and micro-CT scans after two months were taken as the gold standard.

**Results:**

The DM results show dentinal crack formation in 47% of the samples following root-end resection and in 87% following ultrasonic preparation. After the ultrasonic preparation, no existing crack propagated to a complete crack, but new cracks were formed. MTA and super-EBA had no effect on crack formation. The Spearman correlation coefficient between OCT and DM was 0.186 (very weak correlation; *p* = 0.015). Sensitivity and specificity in comparison to micro-CT were 0.50 and 0.55 in OCT and 1.00 and 0.35 in DM, respectively. McNemar's test showed a significant difference between OCT and DM (*p* < 0.05).

**Conclusion:**

Apical resection and ultrasonic preparation could form dentinal cracks. OCT and DM showed different detection frequencies of cracks with very weak correlation. DM showed superior sensitivity compared with OCT.

## 1. Introduction

The prognosis of surgical endodontics depends on several factors. The improvements of microsurgical procedures, such as the use of a dental operative microscope, microinstruments, ultrasonic tips, and the use of more biocompatible obturation materials, have increased the success rate of microsurgical endodontics [1]. However, a dentinal defect is one of the factors that can adversely affect the outcome of endodontic microsurgery; according to a recent prospective study on endodontic microsurgery, a superior outcome was obtained for an intact root compared with a root with a dentinal defect at one-year and three-year postoperative follow-ups [2].

The causes of dentinal defects during microsurgical endodontics have been previously investigated, focusing on the effect of ultrasonic root-end preparation [2–4]. In the resected root-end, there were significantly more cracks after ultrasonic root-end preparation than after root resection alone [3]. The power setting of an ultrasonic device has an effect on crack formation, showing more cracks with a high-frequency setting compared to a low-frequency setting [3]. Compared to bur cavity preparation, ultrasonic cavity preparation showed a significantly higher incidence of crack formation in the walls of root-end cavities [5]. Moreover, preexisting dentinal defects can be propagated by ultrasonic root-end preparation, as revealed by a surgical operating microscopic inspection before and after ultrasonic preparation [4]. However, association of ultrasonic root-end preparation with crack formation and/or propagation still seems controversial, since several studies failed to detect any significant influence of the use of ultrasonic devices on the formation and/or propagation of cracks in resected root ends [6, 7].

Optical coherence tomography (OCT) has been shown previously to be a successful diagnostic method to observe enamel cracks and dentinal cracks [8]. It was first used in ophthalmology, followed by different medical and dental fields [9]. It can observe cross-sectional images of subsurface microstructures. Cross-sectional images are generated by multiple axial measurements of echo time delays called an A-scan. Multiple A-scans generate a B-scan, forming a two-dimensional cross-sectional image. The tissue changes visualized through grayscale differ according to the optical properties of the tissues [8, 10]. Under OCT, crack lines in the dentin should appear as a white line due to the differences in refractive indexes between air or water and dentin. The refractive index of dentin is calculated as 1.55 [11], while the refractive index of air and water is 1.00 and 1.33, respectively [12].

Mineral trioxide aggregate (MTA) has been used as root-end filling materials of choice due to its excellent sealing ability and biocompatibility [13, 14]. MTA has a long setting time of approximately 2 h and 45 min, and its compressive strength increases over 21 days [15]. MTA shows setting expansion due to water uptake and formation of apatite crystals at the MTA-dentin interference [7]; the expansion during and/or after setting could carry the possibility of inducing force generation leading to crack propagation [16], although the expansion can be considered as a mechanism of its excellent sealing ability [7]. The condensation force might also have some effect on the dentin. However, influence of those forces on root crack formation and/or propagation has not yet been addressed.

This study is aimed at determining (1) the effect of root-end resection, ultrasonic preparation, and root-end filling on the incidence of crack formation and propagation in the resected root end, by using a digital microscope (DM) and OCT and (2) the accuracy of OCT on the detection of cracks. The hypothesis of this study was that there is no effect of root-end resection, ultrasonic root-end cavity preparation, and root-end filling on crack formation and/or propagation. The second hypothesis was that there is no relation between OCT and DM.

## 2. Materials and Methods

### 2.1. Teeth Selection and Preparation

Thirty extracted human mandibular incisors that were free of caries, restoration, and root canal treatments were selected randomly from teeth stored in a container with distilled water in the endodontic laboratory at the Tokyo Medical and Dental University (age, sex, and race of the patients were unknown). This study was approved by the Institutional Review Board of the Tokyo Medical and Dental University (No. D2014-033). Radiographs were taken using a digital X-ray system (Dentnavi, Yoshida, Tokyo, Japan) to select mandibular incisors with single canal. The samples were stored in phosphate-buffered saline (PBS; Nippon Gene, Tokyo, Japan) inside a water bath (Thermax TM-2A, AS ONE, Osaka, Japan) at a temperature of 37.0°C for one week before preparation.

The root of the samples was covered with one layer of aluminum foil and embedded in acrylic resin (Unifast III, GC, Tokyo, Japan). After that, the samples with the foil layer were removed, and hydrophilic vinyl polysiloxane impression material (Exafine regular type, GC) was placed around the root to simulate the periodontal ligament. The crown was sectioned 2 mm above the proximal cementoenamel junction for coronal access using a water-cooled low-speed saw (IsoMet, Buehler, Lake Bluff, IL, USA).

Root canal instrumentation was performed using ProTaper NEXT (PTN) rotary instruments (Dentsply Sirona, Ballaigues, Switzerland) driven by an endodontic motor (X-Smart Plus, Dentsply Sirona). Following coronal flaring with ProTaper SX instrument (Dentsply Sirona), the canal was instrumented with ProTaper NEXT X1, X2, and X3 instruments with a rotary speed of 300 pm and 200 g/cm torque using brushing motion. The canal was irrigated with 2 ml of 6% NaOCl (Purelox, Oyalox, Tokyo, Japan) after every instrument change. After instrumentation, the prepared canal received a final irrigation sequence of 5 ml of 14% ethylenediaminetetraacetic acid (Showa Yakuhin Kako, Tokyo, Japan) for 2 min and dried using paper points, and then, obturation was performed with gutta-percha points (GC) and sealer (AH Plus, Dentsply Sirona) using the cold lateral condensing technique.

Root-end resection, root-end cavity preparation, and root-end filling were done using dental operating microscope (ManiScope, Mani, Tochigi, Japan) under LED light at 100 V and 130 W with total magnification of 15x. Apical root resection was performed by removing apical 3 mm at 90 degrees to the long axis of the root with a #701 crosscut fissure bur (Dentsply Sirona) in a high-speed handpiece (Morita, Kyoto, Japan) with water coolant. A retrograde cavity was prepared using an ultrasonic device (Morita, Kyoto, Japan) and an ultrasonic retrotip (Solfy Retro Tip R2, Morita) to a 3 mm depth with a power setting of 5 with water coolant.

Samples were divided randomly into three groups (*n* = 10, each), according to the material for root-end filling; group MTA and group EBA were filled with white ProRoot MTA (Dentsply Sirona) and super-EBA fast set (Harry J. Bosworth, Skokie, IL, USA), respectively, whereas no obturation was done in the control group. After drying the cavity with air spray, the cavity was filled for groups MTA and EBA. MTA was mixed in a 3 : 1 powder-to-water ratio using sterile water and was incrementally placed into the cavity. The material was condensed with a plugger (Sacred, Sialkot, Pakistan) and burnished with a ball burnisher (Sacred, Sialkot, Pakistan) to remove the excess. Super-EBA was mixed according to the manufacturer's direction in a 4 : 1 powder-to-liquid ratio until it had a putty-like consistency and incrementally placed and burnished in the same manner as in the MTA group. The samples were kept in PBS at 37.0°C.

### 2.2. Micro-CT Imaging as the Reference Standard

Each sample was scanned using a microfocus X-ray computed tomography (micro-CT) (inspeXio SMX-100CT, Shimadzu, Kyoto, Japan) twice: before any treatment was done to exclude existing fractures or cracks and after two months of the root-end resection filling. Exposure parameters were set at 80 kV and 130 *μ*A with a voxel size of 20 *μ*m.

### 2.3. OCT Scanning

Scanning of each sample was performed using a swept-source OCT (Santec OCT-2000, Santec Co., Komaki, Japan) immediately following the root-end resection, ultrasonic preparation, and root-end filling. Follow-up scanning was also done at two weeks, one month, and two months. This system consists of swept-source HSL (high-speed laser source), a Mach-Zehnder-type interferometer, inner vision OCT imaging software, a microscopic type probe, and an adjustable stage. The light beam is projected from the probe parallel to the long axis of the sample. The OCT system has a 1310 nm center wavelength and a 20 kHz sweep rate. Laser power was <10 mW, which is within the American National Standards Institute limit. The axial resolution of this system in air is 11 *μ*m, which is equal to 7 *μ*m in dental tissue with a refractive index of about 1.5. The size of each image was 400 × 400 × 1000 voxels, which corresponded to 7.00 × 7.00 × 7.48 mm.

### 2.4. Digital Microscope Observation

Each sample was observed using a digital microscope (DM; VH-8000, Keyence, Osaka, Japan) with 40x magnification, under an external LED light source (Iris, Iris Ohyama Group, Kakuda, Japan) at 100 V and 14 W. Methylene blue dye (Weldeck, Munster, Germany) was applied with a microbrush (Micro Applicator PICO, B.S.A. Sakurai, Nagoya, Japan) to the apical surface prior to the DM observation, rinsed off with a saline solution (Otsuka Pharmaceutical, Tokushima, Japan), and dried with gauze (Hakujuji Medical Product, Tokyo, Japan). The DM observation was done immediately following the apical root resection, ultrasonic preparation, and root-end filling. Follow-up scanning with DM was done at the same time periods as the OCT scanning.

### 2.5. Dentinal Crack Evaluation

For OCT, cross-sectional images of the apical part were constructed using Amira software (Amira 5.6, FEI Visualization Sciences Group, Burlington, MA, USA) to form animated videos (20 s, 600 frames). OCT videos of the apical surface after the root-end resection, ultrasonic preparation, and root-end filling were compared with DM images. The DM images and OCT videos were imported randomly in the Microsoft PowerPoint program (Microsoft Office 2010; Microsoft Corp, Seattle, WA, USA) on a computer (LG E2250; LG Electronics, UK). The evaluation was done by one evaluator (Y.I.) who has experienced as a dentist and an OCT user in the Department of Pulp Biology and Endodontics, Tokyo Medical and Dental University. Prior to evaluation, the evaluator was trained by examining OCT animations and DM images that were not included in this study for fifteen minutes.

**Figure 1:**
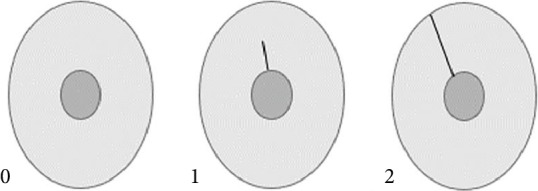
Schematic representation of the grading scale of crack: intact (0), partial crack (1), and complete crack (2).

A dentinal crack was graded as (0) intact; (1) a partial dental crack, when it extended from the canal wall to the dentin; and (2) a complete dentinal crack, when it extended from the canal orifice to the cementum (Figure 1). The propagation of a dentinal crack, when a partial crack extended into a complete crack, was evaluated. This experiment was a single blinded test; the images were placed in PowerPoint by B.R. and given blindly to the evaluator.

### 2.6. Statistical Analysis

The statistical analysis was performed using SPSS statistics v 22 software (SPSS, Chicago, IL). The sensitivity, specificity, positive predictive value (PPV), and negative predictive value (NPV) were determined for OCT and DM in relation to micro-CT. The Spearman correlation coefficient was used to compare the frequency of crack formation. McNemar's test was used to check if there is a significance in the frequency of crack detection between DM and OCT.

## 3. Results

**Figure 2:**
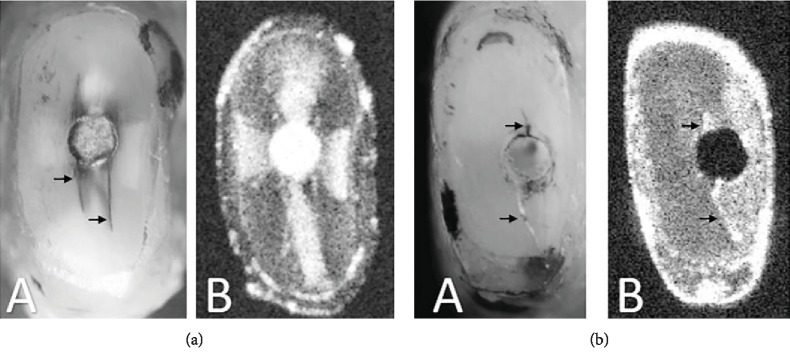
Representative images showing the same surface by DM (A) and OCT (B). (a) MTA group after two months of obturation. (b) Control group immediately after ultrasonic preparation. Arrows: crack lines.

Figures 2(a) A and 2(b) A show the representative DM images showing crack formation after root-end resection. DM observation detected crack formation in 47% and 87% of samples after the root-end resection and ultrasonic root-end cavity preparation, respectively. Forty percent of the resected surfaces had new dentinal cracks after the ultrasonic preparation, while only 3% had dental cracks that propagated from a partial crack to a complete crack.

Figures 2(a) B and 2(b) B present the representative OCT images with their corresponding DM images. The differences in contrast in the dentin were clear in Figure 2(a) B; the crack line is visualized as a white line caused by the high backscattered intensities, as shown in Figure 2(b) B. OCT detected crack formation in 40% of the samples after the root-end resection and in 30% of the samples after the ultrasonic root-end cavity preparation. The Spearman correlation coefficient between OCT and DM after 170 observation of the three groups in different time intervals was 0.186 (very weak correlation) with *p* value = 0.015. In addition, McNemar's test showed a significant difference between DM and OCT with *p* value < 0.05.

The Spearman correlation coefficient for the frequency of crack detection by DM showed no significant differences in any of intergroup comparisons within the same time point and inter-time-point comparisons within the same group, with *p* > 0.05. The Spearman correlation coefficient immediately after ultrasonic preparation and after two months were 0.61, 1.00, and 0.82 for the control, group MTA, and group EBA, respectively.

The prevalence of crack formation observed by micro-CT after two months of the microsurgical procedure was 30% for the three groups. MTA and EBA did not affect the frequency of cracks (20% vs. 30% at 2 months, respectively) with a strong spearman correlation coefficient (0.71, *p* < 0.05).  Table 1 shows the overall sensitivity, specificity, PPV, and NPV of DM and OCT for the detection of dentinal crack formation (score 0 vs. scores 1 and 2).

## 4. Discussion

In this study, the dentinal crack formation during endodontic root-end surgery was evaluated using two diagnostic techniques, DM with methylene blue dye and OCT. The cracks were clearly observed with DM after root-end resection, and the formation of a new crack was detected after the ultrasonic root-end cavity preparation while the existing partial cracks did not propagate into complete cracks. The results may support the view that ultrasonic root-end preparation causes formation and/or propagation of dentinal cracks, which is in line with several studies that used different types of microscopes as a diagnostic method in detecting dental cracks [3, 5]. However, some studies did not show any crack formation after ultrasonic retropreparation [6, 17]. Also, the present findings contrast with a previous study, which illustrated that ultrasonic preparation propagates preexisting cracks while remaining safe to be used on an intact tooth. This finding was obtained by using surgical operating microscope and light-emitting diode microscope diagnostic probe light [4]. The difference between studies could be due to differences of experimental condition and suggests that the crack detection could be technique sensitive.

In the present study, samples were placed in PBS solution in water bath at 37.0°C to mimic oral condition and to prevent desiccation of dentin which may lead to dental cracks. In addition, impression materials were placed as PDL simulation to minimize the crack lines caused by rotary instruments by allowing limited free movement [18].

Under DM, the magnification together with the use of methylene blue dye and an external light showed superficial craze line-like structure that could include microcracks that were not detected by micro-CT, leading to an increased number of false positives and a decreased specificity and PPV.

MTA and super-EBA are commonly used root-end filling materials, and this study evaluated the effect of these materials on crack formation and/or propagation because setting expansion and condensation force of root-end filling materials could carry the possibility of generating forces that cause crack formation/propagation. In this study, there were no significant differences among the control group, group MTA, and group EBA in crack formation in all the follow-up evaluations. Thus, under present experimental conditions, root-end filling was not a factor that causes crack formation and/or propagation.

The OCT system is a noninvasive, nonradiative imaging technique, which constructs cross-sectional images of internal biological structures and materials. The diagnostic ability of OCT enabled ophthalmologist to visualize different layers of retina with no direct contact. It is also used as diagnostic method for coronary atherosclerosis with a modified catheter used as an optical fiber [19]. Gradually, the uses of OCT extended to other fields such as dermatology [20], neurology, [21], and dentistry. In dentistry, the OCT system is able to diagnose occlusal, proximal, and cervical caries [8]. It is also used to compare different adhesive materials in gap formations under composite restorations [22, 23]. Enamel crack detection [10], vertical root fractures, [24], and dental composite cracks [25] were diagnosed previously using OCT.

Under the present experimental condition, OCT overall showed a lower performance in detecting dentin crack lines compared with DM. In particular, sensitivity and NPV of OCT were lower than those of DM. Micro-CT was used as a reference standard. Up to our knowledge, DM results have not yet been compared to micro-CT results before, while micro-CT has been used as a reference standard to evaluate OCT results in several studies [26, 27]. The Spearman correlation between DM and OCT was low in addition to the low sensitivity and specificity of OCT. McNemar's test showed a significant difference between DM and OCT. One reason for the lower performance of OCT may be that the resolution of OCT was not high enough, resulting in somewhat blurred images without clear borderlines. The use of noise filter(s) could improve the reliability of OCT, since a recent study has reported that the use of an edge enhancement filter that augments the contrast between the crack line and background improves the detection of enamel crack formation [28].

Moreover, optical properties of dentin could affect the ability of OCT to detect dentinal cracks. Unlike enamel, the light propagation of dentin is not isotropic. The light is scattered mainly by the tubules, and their orientation affects light scattering [11]. Light reflection occurs more in peritubular dentin because of its high refractive index compared to the tubules and intratubular dentin [29]. The low contrast between the dentin surrounding the canal area and the crack line made it difficult to recognize the formation of partial dentinal cracks.

In summary, the first hypothesis that “there is no effect of root-end resection, ultrasonic preparation, and root-end filling materials on crack formation and/or propagation neither immediately nor after follow-ups” was partly rejected, since DM detected an increased incidence of crack formation after apical resection and ultrasonic root-end preparation. The second hypothesis that there is no relation between OCT and DM was accepted.

## 5. Conclusions

DM observation with methylene blue staining demonstrated that ultrasonic root-end preparation induced crack formation whereas the type of root-end filling material (MTA or super-EBA) showed no effect on dentinal crack formation over two months. OCT and DM showed different detection frequencies of cracks with very weak correlation. DM showed superior sensitivity compared with OCT.

## Figures and Tables

**Table 1:** Overall sensitivity, specificity, positive predictive value (PPV), and negative predictive value (NPV) of OCT and DM compared to micro-CT in detecting crack formation after two months of root-end resection and filling.

	Sensitivity	Specificity	PPV	NPV
OCT	0.50	0.55	0.36	0.69
DM	1.00	0.35	0.43	1.00

## Data Availability

The datasets used and/or analyzed during the current study are available from the corresponding author on reasonable request.

## References

[1] Kim S., Kratchman S. (2006). Modern endodontic surgery concepts and practice: a review. *Journal of Endodontics*.

[2] Tawil P. Z., Saraiya V. M., Galicia J. C., Duggan D. J. (2015). Periapical microsurgery: the effect of root dentinal defects on short- and long-term outcome. *Journal of Endodontics*.

[3] Layton C. A., Marshall J. G., Morgan L. A., Baumgartner J. C. (1996). Evaluation of cracks associated with ultrasonic root-end preparation. *Journal of Endodontics*.

[4] Tawil P. Z. (2016). Periapical microsurgery: can ultrasonic root-end preparations clinically create or propagate dentinal defects?. *Journal of Endodontics*.

[5] Abedi H. R., van Mierlo B. L., Wilder-Smith P., Torabinejad M. (1995). Effects of ultrasonic root-end cavity preparation on the root apex. *Oral Surgery, Oral Medicine, Oral Pathology, Oral Radiology, and Endodontology*.

[6] Beling K. L., Marshall J. G., Morgan L. A., Baumgartner J. C. (1997). Evaluation of cracks associated with ultrasonic root-end preparation of gutta- percha filled canals. *Journal of Endodontics*.

[7] Torabinejad M. (2014). *Mineral trioxide aggregate: properties and clinical applications*.

[8] Shimada Y., Sadr A., Sumi Y., Tagami J. (2015). Application of optical coherence tomography (OCT) for diagnosis of caries, cracks, and defects of restorations. *Current Oral Health Reports*.

[9] Abtahian F., Jang I. K. (2012). Optical coherence tomography: basics, current application and future potential. *Current Opinion in Pharmacology*.

[10] Imai K., Shimada Y., Sadr A., Sumi Y., Tagami J. (2012). Noninvasive cross-sectional visualization of enamel cracks by optical coherence tomography *in vitro*. *Journal of Endodontics*.

[11] Hariri I., Sadr A., Shimada Y., Tagami J., Sumi Y. (2012). Effects of structural orientation of enamel and dentine on light attenuation and local refractive index: an optical coherence tomography study. *Journal of Dentistry*.

[12] Zhou Y., Chan K. K., Lai T., Tang S. (2013). Characterizing refractive index and thickness of biological tissues using combined multiphoton microscopy and optical coherence tomography. *Biomedical Optics Express*.

[13] Aqrabawi J. (2000). Sealing ability of amalgam, super EBA cement, and MTA when used as retrograde filling materials. *British Dental Journal*.

[14] Adamo H. L., Buruiana R., Schertzer L., Boylan R. J. (1999). A comparison of MTA, super-EBA, composite and amalgam as root-end filling materials using a bacterial microleakage model. *International Endodontic Journal*.

[15] Torabinejad M., Hong C. U., McDonald F., Ford T. P. (1995). Physical and chemical properties of a new root-end filling material. *Journal of Endodontics*.

[16] Ørstavik D. (2014). Endodontic filling materials. *Endodontic Topics*.

[17] Calzonetti K. J., Iwanowski T., Komorowski R., Friedman S. (1998). Ultrasonic root end cavity preparation assessed by an in situ impression technique. *Oral Surgery, Oral Medicine, Oral Pathology, Oral Radiology and Endodontology*.

[18] Karataş E., Gündüz H. A., Kırıcı D. Ö., Arslan H., Topçu M. Ç., Yeter K. Y. (2015). Dentinal crack formation during root canal preparations by the twisted file adaptive, ProTaper Next, ProTaper Universal, and WaveOne instruments. *Journal of Endodontics*.

[19] Jang I. K., Bouma B. E., Kang D. H. (2002). Visualization of coronary atherosclerotic plaques in patients using optical coherence tomography: comparison with intravascular ultrasound. *Journal of the American College of Cardiology*.

[20] Pierce M. C., Strasswimmer J., Park B. H., Cense B., de Boer J. F. (2004). Advances in optical coherence tomography imaging for dermatology. *Journal of Investigative Dermatology*.

[21] Pulicken M., Gordon-Lipkin E., Balcer L. J., Frohman E., Cutter G., Calabresi P. A. (2007). Optical coherence tomography and disease subtype in multiple sclerosis. *Neurology*.

[22] Makishi P., Shimada Y., Sadr A., Tagami J., Sumi Y. (2011). Non-destructive 3D imaging of composite restorations using optical coherence tomography: marginal adaptation of self-etch adhesives. *Journal of Dentistry*.

[23] Bakhsh T. A., Sadr A., Shimada Y., Tagami J., Sumi Y. (2011). Non-invasive quantification of resin–dentin interfacial gaps using optical coherence tomography: validation against confocal microscopy. *Dental Materials*.

[24] Shemesh H., van Soest G., Wu M. K., Wesselink P. R. (2008). Diagnosis of vertical root fractures with optical coherence tomography. *Journal of Endodontics*.

[25] Braz A. K., Kyotoku B. B., Braz R., Gomes A. S. (2009). Evaluation of crack propagation in dental composites by optical coherence tomography. *Dental Materials*.

[26] Ding J., Ebihara A., Watanabe S. (2014). Application of optical coherence tomography to identify pulp exposure during access cavity preparation using an Er: YAG laser. *Photomedicine and Laser Surgery*.

[27] Iino Y., Ebihara A., Yoshioka T. (2014). Detection of a second mesiobuccal canal in maxillary molars by swept-source optical coherence tomography. *Journal of Endodontics*.

[28] Kim J. M., Kang S. R., Yi W. J. (2017). Automatic detection of tooth cracks in optical coherence tomography images. *Journal of Periodontal & Implant Science*.

[29] Kienle A., Forster F. K., Diebolder R., Hibst R. (2003). Light propagation in dentin: influence of microstructure on anisotropy. *Physics in Medicine & Biology*.

